# Cinnamon (*Cinnamomum cassia*) hot water extract improves inflammation and tight junctions in the intestine in vitro and in vivo

**DOI:** 10.1007/s10068-023-01292-3

**Published:** 2023-03-27

**Authors:** Tae gwon Park, Yu Rim Kim, Soo-yeon Park, Kwanyong Choi, Kyeong Jin Kim, Ji Yeon Kim

**Affiliations:** 1 Department of Food Science and Technology, Seoul National University of Science and Technology, 232, Gongneung-Ro, Nowon-Gu, Seoul, 01811 Republic of Korea; 2 Department of Nano Bio Engineering, Seoul National University of Science and Technology, 232, Gongneung-Ro, Nowon-Gu, Seoul, 01811 Republic of Korea

**Keywords:** *Cinnamomum cassia*, Cinnamon, Inflammatory bowel disease, Tight junctions, Anti-inflammatory effect

## Abstract

The natural byproduct *Cinnamomum cassia* was widely used in ancient Asia to cure disease because of its various pharmacological effects. Despite its ethnomedicinal benefits, few studies on the intestinal anti-inflammatory effect of *C. cassia* have been reported. Therefore, this study aimed to evaluate the potential beneficial effects of *C. cassia* on the intestine in vitro and in vivo. Herein, the effects of cinnamon hot water extract (CWE) on tight junction (TJ) barrier function, transepithelial electrical resistance, and mRNA expression were confirmed in Caco-2 cells. The CWE treatment groups showed significantly enhanced cell permeability, proinflammatory cytokine mRNA expression, and TJ expression. CWE-treated mice showed an improved histological index and decreased cytokine concentrations compared with those of colitis model mice. These results suggest that CWE alleviated inflammatory damage and improved the TJ barrier, indicating that CWE may be used as a functional food to improve intestinal health.

**Supplementary Information:**

The online version contains supplementary material available at 10.1007/s10068-023-01292-3.

## Introduction

Inflammatory bowel disease (IBD), an acute inflammatory disease of the large intestine with an unclear mechanism, imposes a large burden on patients with IBD, the number of which has been growing over the past 50 years (Gasparetto and Guariso, 2013). Ulcerative colitis (UC) and Crohn’s disease (CD) can trigger leaky gut syndrome, which causes immune cells such as lymphocytes, macrophages, and neutrophils to become activated, assemble at sites of inflammatory reactions, and promote intestinal inflammation by overproducing TNF-α, IL-1β, IL-6 and IL-23 (Medzhitov and Janeway Jr, 1997; Wu et al., 2014). Intestinal epithelial cells, which are the outpost of defense against the invasion of harmful external substances, form the intestinal epithelium and consist of various cell types, such as intestinal cells, goblet cells, and immune cells (Romier-Crouzet et al., 2009). Impairment of gastrointestinal barrier function consequently has clinical and nutritional consequences, which result in conditions such as malnutrition, inflammation, chronic disorders, and loosening of tight junctions (TJs), which can lead to leaky gut syndrome. The collapse of TJs and intestinal inflammation cause various diseases, which can be prevented or relieved by enhancing the function of the bowel. Where these disorders are noted, phytochemicals and other nutritional supplements have proven beneficial and effective in improving TJ protein and function (Ghosh et al., 2020).

Cinnamon has been classified into 250 species worldwide (Kumar et al., 2019). Cinnamon is a natural remedy without side effects that has been used since ancient times. According to prior studies, cinnamon exerts antioxidant, antimicrobial, anticancer, and anti-inflammatory effects (Ruwizhi and Aderibigbe, 2020) with no signs of toxicity (Hoskins, 1984). Moreover, cinnamon inhibits the growth of *Helicobacter pylori*, thus reducing the risk of gastritis, duodenal ulcers, and gastric lymphoma (Gruenwald et al., 2010). This finding suggests that cinnamon is a strong candidate for IBD treatment due to its function against proinflammatory cytokines. Many studies have investigated the use of different cinnamon species to improve intestinal health.

However, reports on the ability of *Cinnamomum cassia* Presl, a cinnamon species, to improve intestinal health are limited. *C. cassia* is a tropical aromatic evergreen tree belonging to the Lauraceae family that is native to China and Southeast Asian countries. Researchers have sought to examine the pharmacological aspects of *C. cassia*, and almost 160 chemical substances from *C. cassia* have been identified (Zhang et al., 2019). Cinnamon contains high levels of bioactive compounds such as cinnamic acid (CA), cinnamic aldehyde, cinnamic alcohol, coumarin, and eugenol, which have been proven to have beneficial health effects (Kim and Kim, 2019). Among them, CA plays a role as a key component of *C. cassia* and *Panax ginseng* and as a dietary ingredient (Ruwizhi and Aderibigbe, 2020). In addition, CA is the source of hydroxy and methoxy derivatives, known as phenylpropanoic acids, derived from food components (Rychlicka et al., 2021).

Thus, the purpose of our research was to assess the anti-inflammatory effects of *C. cassia* and CA and their ability to enhance TJ function in a dextran sulfate sodium (DSS)-induced colitis mouse model and Caco-2 human intestinal epithelial cells.

## Materials and methods

### Materials

Dulbecco’s modified Eagle’s medium (DMEM), fetal bovine serum (FBS), and Dulbecco’s phosphate-buffered saline (DPBS) were produced by Biowest (Nuaillé, Cholet, France). Nonessential amino acids (NEAAs) and penicillin–streptomycin were purchased from Gibco (Rockville, MD, USA). A Cell Counting Kit-8 (CCK-8) assay kit and 10% formalin were purchased from Sigma‒Aldrich (St. Louis, MO, USA). ELISA kits for IL-1β, IL-6, and TNF-α were purchased from R&D Systems (Minneapolis, MN, USA), and DSS (Mw: 36,000–50,000) was purchased from MP Biomedical (Solon, OH, USA).

### Preparation of CWE

The *C. cassia* used in this experiment was kindly provided by the Cinnamon Lab (Seoul, Korea). Briefly, CWE was prepared by adding 10 volumes of purified water to 50 kg of cinnamon and incubating the mixture at 121–130 °C for 8 h. The extracted cinnamon was filtered through 1 μm filter paper, and then 6 volumes of purified water were added for 4 h of further treatment at 121–130 °C. The mixture was concentrated under reduced pressure at low temperature. After adding dextrin, dissolution was carried out at 65–85 °C for 40 min. Sterilization was performed at 65–85 °C, followed by drying and cooling using a high-temperature instantaneous dryer to obtain the cinnamon extract. The amount of cinnamic acid (CA) within the CWE was analyzed by HPLC. The analytical conditions are listed in Table S1.

### Cell culture

Caco-2 cells were obtained from the Korean Cell Line Bank (Seoul, Korea). The cells were cultivated in DMEM supplemented with 10% FBS, 100 U/mL penicillin, 100 μg/mL streptomycin, and 1% NEAAs at 5% CO_2_ and 37 °C in an incubator. The Caco-2 cells were seeded into 6-Transwell® plates (0.4 µm pore size; Corning Costar Corp., NY, USA) at 1 × 10^5^ cells per well. The medium was changed twice a week. Cell viability was determined by CCK assay according to the manufacturer’s guidelines. The absorbance at 450 nm was measured using a microplate reader (PowerWave XS2 Microplate Reader, BioTek, Winooski, VT, USA). Cells at passages 30–40 were used in the experiments.

### Measurement of TJ barrier function

TJ barrier function was measured by determining transepithelial electrical resistance (TEER) in differentiated Caco-2 monolayers. CWE (50 or 100 μg/mL), CA (0.35 μg/mL), and lipopolysaccharide (LPS) (10 μg/mL) were injected into the apical well and incubated overnight with an inflammatory cytokine cocktail in the basal well (IFN-γ and TNF-α, 50 ng/mL; IL-1β, 25 ng/mL; LPS 10 ng/mL). The TEER values were measured three consecutive times 48 h after treatment with the cytokine cocktail using a Millicell® ERS instrument (Millipore, Bedford, MA, USA). The data are presented as a % of the initial value.

### mRNA extraction from Caco-2 cells and qRT‒PCR

Total RNA was isolated from the cultured cells by TRIzol (Life Technologies, Rockville, MD, USA). The Caco-2 cells were lysed with TRIzol reagent, chloroform was added after 5 min, and the solution was mixed briefly with an equivalent volume of isopropanol. cDNA was synthesized from the total RNA using a Transcriptor First-Strand cDNA Synthesis Kit (Hoffmann-La Roche Ltd., Basel, Switzerland). qRT‒PCR was conducted by a LightCycler® 96 system (Hoffmann-La Roche Ltd., Basel, Switzerland). Relative mRNA expression was quantified by using the comparative 2^−ΔΔCT^ method after normalization to the amount of GAPDH. The forward and reverse primer sequences are listed in Table S2.

### Animals

Five-week-old female BALB/c mice were obtained from Orient Bio (Seongnam, Korea) and housed under the following conditions for 7 days: a temperature of 22 ± 2 °C, a relative humidity of 50 ± 10%, a 12 h-12 h light–dark cycle, and a commercial diet and 5% DSS water provided ad libitum. This study was conducted with the approval of the IACUC (KIRAMS 2022-0017) of the Korea Cancer Center Hospital (Seoul, Korea).

### Colitis modeling and administration of CWE

The mice were randomly divided into the following 5 groups (*n* = 8 animals/group): the control, 5% DSS-induced colitis, DSS + CWE (100 mg/kg B.W.), DSS + CWE (500 mg/kg B.W.), and DSS + CA (1.7 mg/kg B.W.) groups. Mice in the CWE and CA groups were administered the experimental materials by oral gavage at the same time every day from days 1 to 21. Body weight was recorded every 3 days. Colitis was induced in the mice by providing drinking water mixed with 5% DSS for one week, from days 15 to 21. During the administration of DSS, body weight, stool consistency, blood in the stool, and anal bleeding were evaluated by a method suggested by Cooper et al. to determine the disease activity index (DAI) (Cooper et al., 1993). Detailed information on the DAI is provided in the supplementary materials in Table S3. Colon length was measured, and the colon tissue was separated and used to determine cytokine concentrations.

### Histological analysis

Distal colon tissue was fixed in 10% formaldehyde, embedded in paraffin blocks, and cut to a thickness of 4 μm. The sections were stained with hematoxylin & eosin (H&E). To analyze colon mucosal proliferation, the colon mucosa was photographed at a magnification of × 100 and used to determine the thickness of the colon mucosa. A histological score was calculated by a method suggested by Dieleman et al. (1998). The specific scoring index is provided in Table S4. Alcian blue and periodic acid-Schiff (AB-PAS) staining was performed to determine the number of goblet cells, which were calculated as the number of goblet cells per 10 villus-crypt units.

### Quantification of inflammatory cytokines in the colon tissue

Colon tissue segments were weighed and homogenized using 350 μL of RIPA buffer and centrifuged at 14,000×*g* for 15 min. The contents of the supernatant, including the TNF-α, IL-1β, and IL-6 levels, were measured with ELISA kits used to calculate the cytokine concentrations according to the manufacturer’s instructions. The cytokine concentration was determined by measuring the absorbance at 450 nm with a PowerWave XS2 microplate reader (Biotek, VT, USA), and the concentration of protein in the supernatant was analyzed with a Bradford assay kit (Bio-Rad, Hercules, CA, USA) following the manufacturer’s guidelines.

### Statistical analysis

All values shown are the mean ± standard deviation (SD). For comparison of the control and cocktail-induced groups in vitro or the control and DSS-induced groups in vivo, Student's* t* test was used. The statistical significance of differences between the sample groups was determined using one-way ANOVA followed by a post hoc Dunnett’s multiple range test. *p* < 0.05 indicated statistical significance. All statistical analyses were performed using SAS 9.4 software (SAS Inst., Cary, NC, USA).

## Results and discussion

### Profiling CA in CWE

**Fig. 1:**
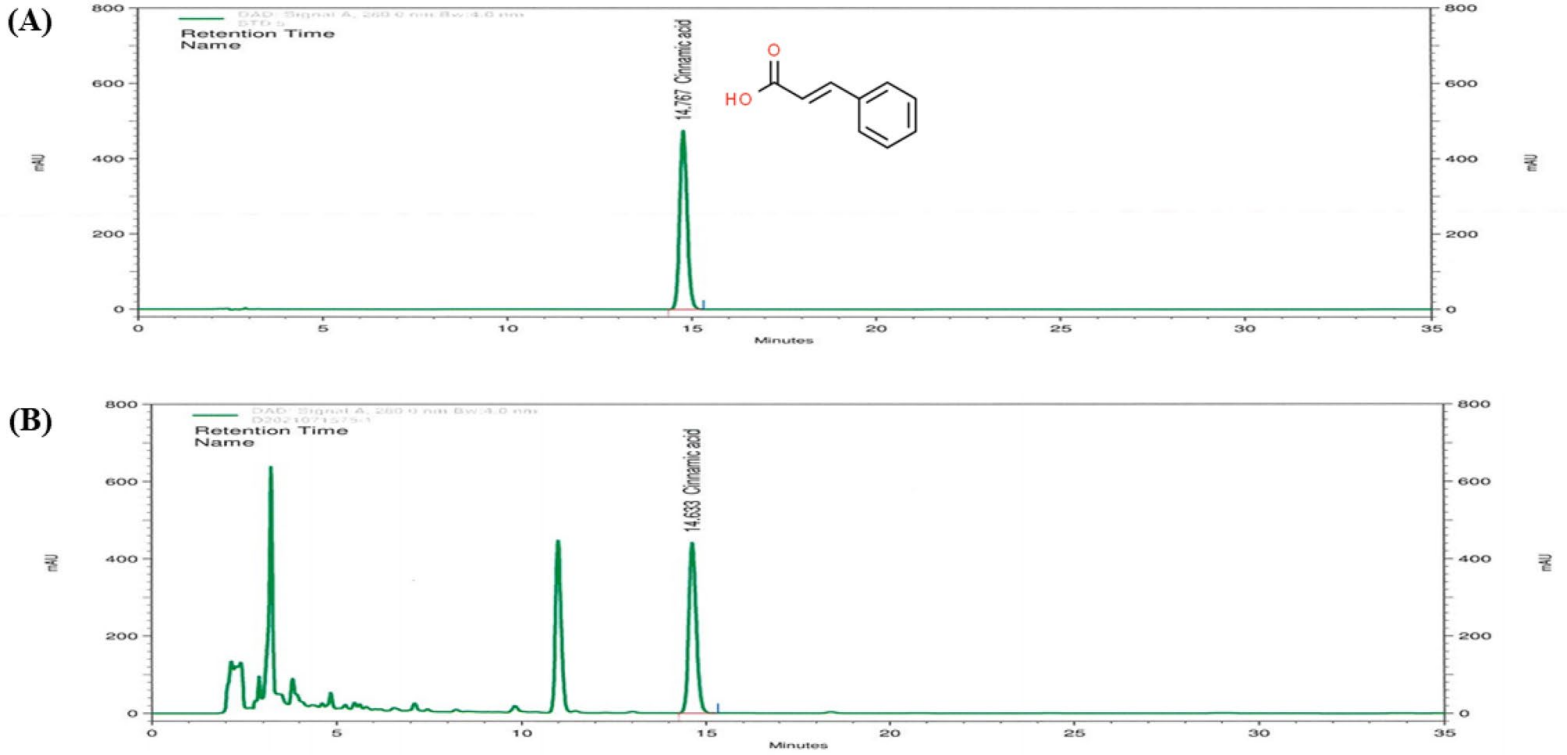
Chromatogram of cinnamic acid in CWE. (**A**) HPLC chromatogram of the standard*.* (**B**) HPLC chromatogram of cinnamic acid in CWE. *CWE* cinnamon hot water extract, *HPLC* high-performance liquid chromatography

CA in CWE was analyzed by HPLC. The chemical structure of CA and the CWE chromatogram are shown in Fig. 1. The total concentration of CA in CWE was 3.5 mg/g. Based on the profiling results, there was no cytotoxic effect under these experimental conditions, as determined by CCK assay (data not shown). According to prior studies, CA, one of the compounds in cinnamon, prevents inflammation and improves damage to intestinal barrier function caused by inflammatory cytokines (Kim and Kim, 2019).

### Effect of CWE treatment on intestinal TJ barrier function in Caco-2 cells

The collapse of the gut epithelial barrier is related to most types of gut inflammation. Caco-2 human intestinal epithelial cells are commonly used to imitate the intestinal epithelial barrier in studies evaluating the effects of samples on TJ permeability and barrier damage. Electrical resistance in the Caco-2 transepithelial layer is proportional to barrier integrity. TEER measurement is an effective method to detect changes in bio/physiological barrier function induced by agents such as LPS, nicotine, and thrombin (Blume et al., 2010), which can be applied to live cells that are observed during the various stages of their expansion and differentiation (Srinivasan et al., 2015). The intestinal barrier function of differentiated Caco-2 cells was compromised by applying an inflammatory cocktail to the basolateral side in this study (Valdez et al., 2020).

**Fig. 2:**
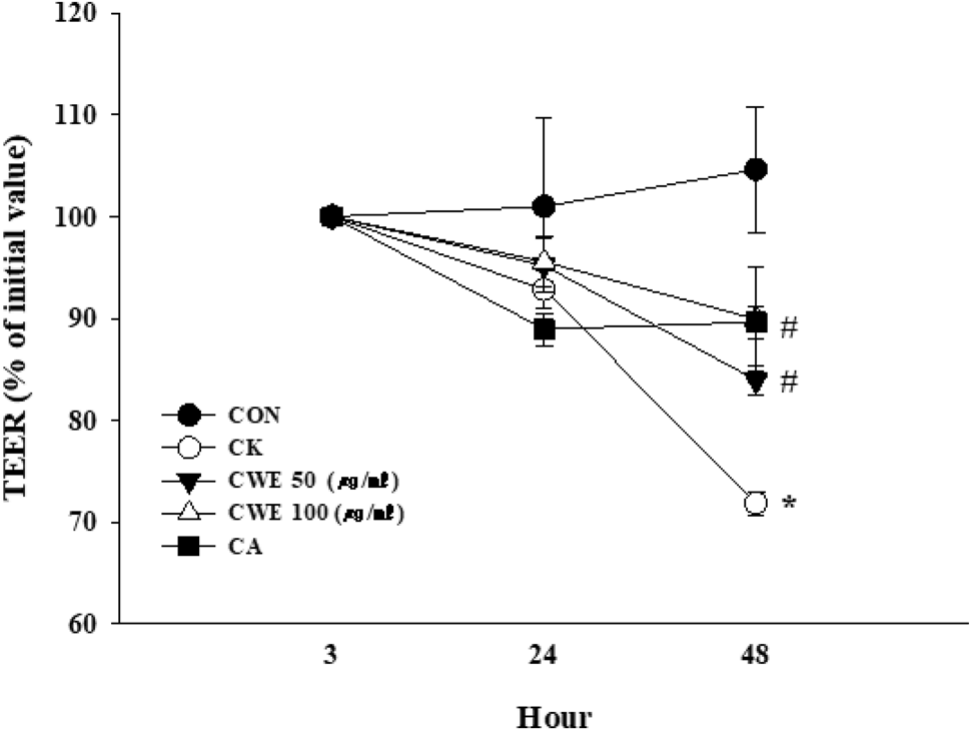
Effects of CWE on transepithelial electrical resistance (TEER) under inflammatory conditions. Samples and LPS were added to the apical side, and a cytokine cocktail was then added to the basolateral side. All values are shown as the mean ± S.D. Asterisks above the bars indicate significant differences between the CON and CK groups (Student’s *t* test; *p* < 0.05). Hash marks above the bars indicate significant differences among the CK-induced groups (Dunnett’s multiple range test; *p* < 0.05). *CON* control, *CK* cytokine cocktail, *CWE 50* CK + 50 µg/mL CWE, *CWE 100* CK + 100 µg/mL CWE, *CA* CK + cinnamic acid

The inflammatory cocktail reduced the TEER value in the CK group and significantly decreased TEER at 48 h compared to that in the control group (*p* = 0.001). Additionally, the TEER value was significantly increased by treatment with 50 and 100 µg/mL CWE and CA compared with that in the CK group (*p* < 0.001, Fig. 2). Both CWE and CA significantly improved barrier integrity compared to that in the CK group at 48 h.

### Effect of CWE on TJ mRNA expression in Caco-2 cells

**Fig. 3:**
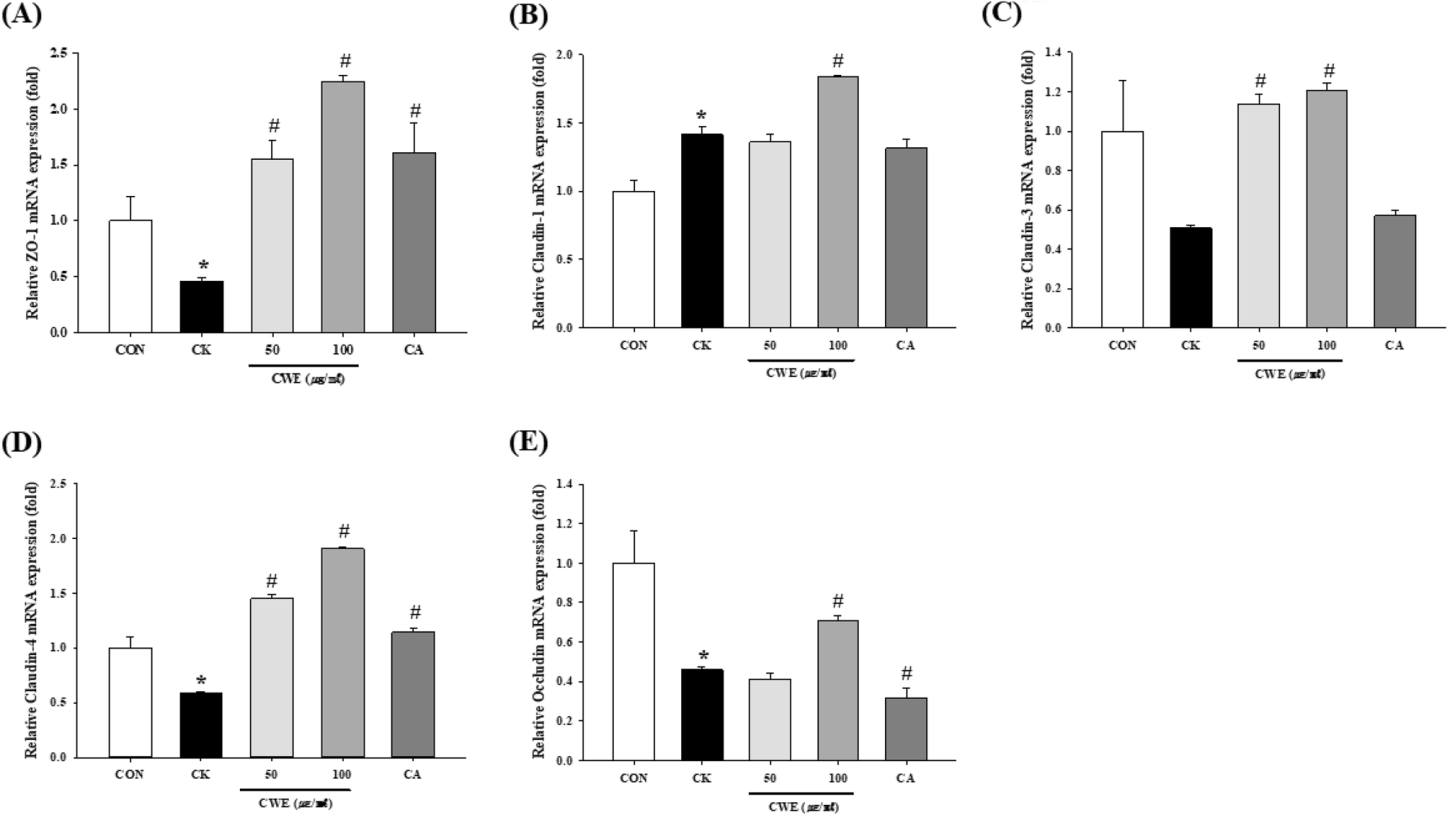
Effect of CWE on TJ mRNA expression in Caco-2 cells under inflammatory conditions. (**A**) ZO-1, (**B**) claudin-1, (**C**) claudin-3, (**D**) claudin-4, and (**E**) occludin expression was measured by qRT‒PCR as described in the Methods. Samples and LPS were added to the apical side, and a cytokine cocktail was then added to the basolateral side. All values are shown as the mean ± S.D. Asterisks above the bars indicate significant differences between the CON and CK groups (Student’s *t* test; *p* < 0.05). Hash marks above the bars indicate significant differences among the CK-induced groups (Dunnett’s multiple range test; *p* < 0.05). *qRT‒PCR* quantitative real-time polymerase chain reaction, *CON* control, *CK* cytokine cocktail, *CWE 50* CK + 50 µg/mL CWE, *CWE 100* CK + 100 µg/mL CWE, *CA* CK + cinnamic acid

The expression of TJ proteins at the mRNA level was then investigated. The treatment of Caco-2 cells with CWE increased the expression of each marker in a dose-dependent manner. In detail, the ZO-1 level increased to the greatest extent (4.9-fold) in cells treated with 100 µg/mL CWE and increased 3.5-fold in cells treated with CA (Fig. 3A). Additionally, the claudin family (claudin-1, claudin-3, and claudin-4) showed significantly increased expression levels after treatment with 100 µg/mL CWE compared to the cytokine cocktail (1.3-fold, 2.3-fold, 3.2-fold, respectively) (Fig. 3B–D). CA significantly increased claudin-4 mRNA expression (2.0-fold) compared to that in the CK group (Fig. 3D), but there was no significant difference in claudin-1 or claudin-3 levels (Fig. 3B, C). In terms of intracellular occludin expression, 100 µg/mL CWE significantly increased occludin expression compared to that in the CK group (1.5-fold) (Fig. 3E). Therefore, both CWE and CA restored barrier disruption through an increase in several TJ proteins, such as ZO-1, occludin, and claudin family members.

The epithelium contains TJs that control the passage of nutrients, molecules, and water through a paracellular pathway via the three essential proteins ZO-1, claudin, and occludin. Structural abnormalities in TJ proteins induce changes in intestinal barrier function. The collapse of the TJ barrier triggers increased barrier permeability and various inflammatory responses. ZO-1 conserves epithelial cell connections, whereas claudin-1 and occludin regulate pericellular permeability in TJs (Landy et al., 2016). Additionally, the deterioration of TJ proteins has been described in IBD patients. We applied a cocktail containing LPS and cytokines to mimic gastrointestinal inflammation. This model was used in several previous studies to mimic inflammatory gastrointestinal conditions (Hwang et al., 2022; Kim et al., 2017; Xu et al., 2021). LPS on the apical side mimics epithelial stimulation by antigens from gram-negative bacteria. Cytokines such as TNF-α, IFN-γ, and IL-1β on the basolateral well have an effect similar to Th-1-type immune activation. TJ abnormalities can be caused by bacterial antigens and Th-1 type immune responses (Al-Sadi et al., 2009; Capaldo and Nusrat, 2009).

### Effect of CWE on proinflammatory mRNA expression in Caco-2 cells

**Fig. 4:**
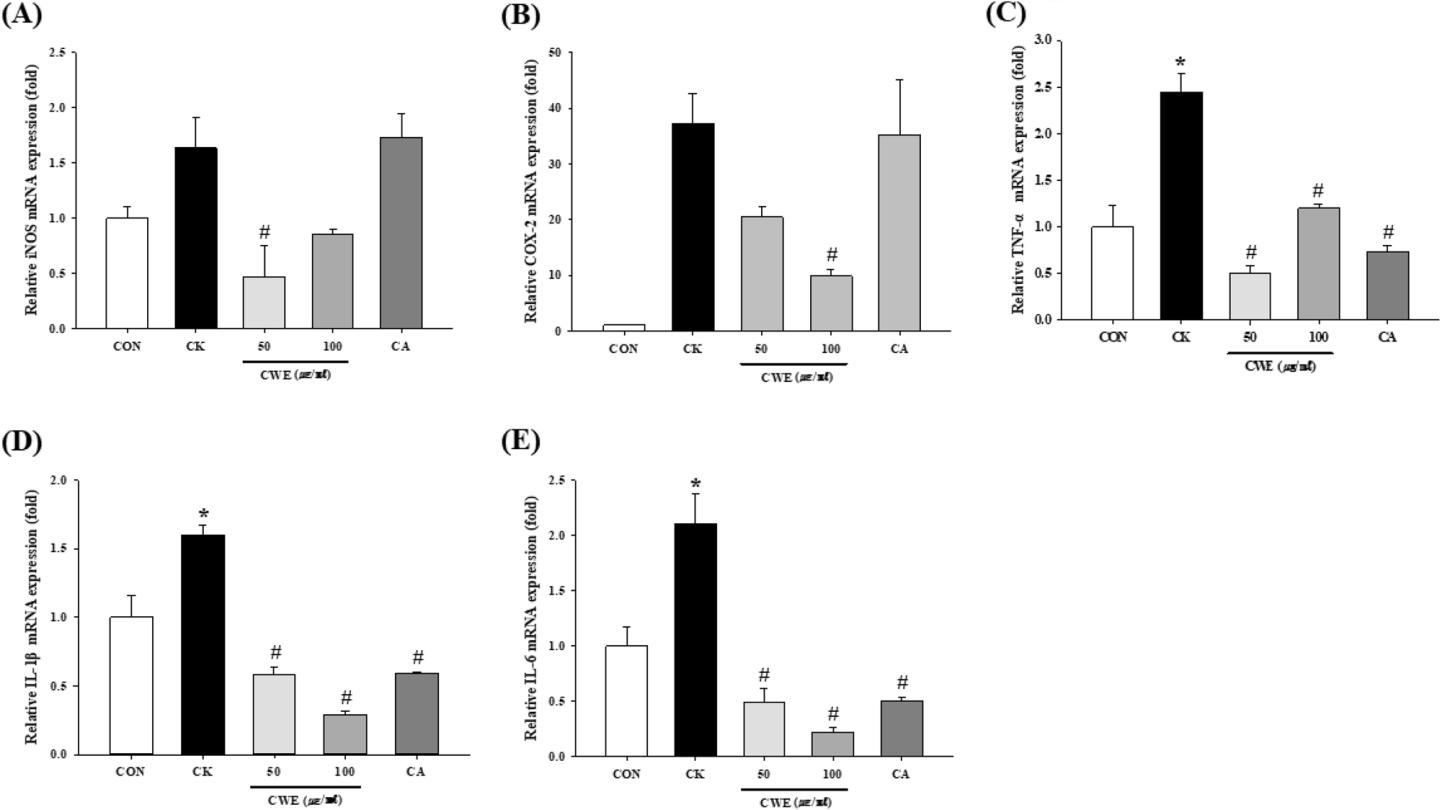
Effect of CWE on inflammatory cytokine mRNA expression in Caco-2 cells under inflammatory conditions. (**A**) iNOS, (**B**) COX-2, (**C**) TNF-a, (**D**) IL-1β, and (**E**) IL-6 expression was measured by qRT‒PCR as described in the Methods. Samples and LPS were added to the apical side, and a cytokine cocktail was then added to the basolateral side. All values are shown as the mean ± S.D. Asterisks above the bars indicate significant differences between the CON and CK groups (Student’s *t* test; *p* < 0.05). Hash marks above the bars indicate significant differences among the CK-induced groups (Dunnett’s multiple range test; *p* < 0.05). *qRT‒PCR* quantitative real-time polymerase chain reaction, *CON* control, *CK* cytokine cocktail, *CWE 50* CK + 50 µg/mL CWE, *CWE 100* CK + 100 µg/mL CWE, *CA* CK + cinnamic acid

CWE decreased the expression of proinflammatory markers in a dose-dependent manner. Specifically, 100 µg/mL CWE reduced the cytokine cocktail-induced inflammatory response by alleviating COX-2, TNF-α, IL-6, and IL-1β expression in Caco-2 cells (0.3-fold, 0.5-fold, 0.1-fold, and 0.2-fold, respectively) (Fig. 4). Caco-2 cells treated with CA showed reduced TNF-α, IL-1β, and IL-6 mRNA levels compared with those in CK-treated cells (0.3-fold, 0.4-fold, and 0.2-fold, respectively) (Fig. 4C–E). However, neither iNOS nor COX-2 expression was significantly different between the CK group and the CA-treated group (Fig. 4A, B).

To imitate inflammatory conditions, Caco-2 cells on the basolateral sides of the coincubation system were exposed to a mixture of cytokines for 24 h. COX-2 is a mediator of the inflammatory response (Im et al., 2021). Its expression is measurable in colonic epithelial cells with IBD, and COX-2 is considered an important mediator of UC (Matuk et al., 2004). The overproduction of NO is catalyzed by the proinflammatory enzyme iNOS, which is produced by microspheres. iNOS induces tissue damage in IBD by causing an imbalance in the intestinal antioxidant defense system (Änggård, 1994). The expression of the cytokines IL-1β and IL-6, which play important roles in the inflammatory phase, correlates with inflammation. TNF-α destroys the mucosal layer by inducing mucosal epithelial cell apoptosis, allowing the inflammatory response to persist.

Based on our results, both CWE and CA exhibited anti-inflammatory activity by decreasing TNF-α, IL-6, and IL-1β mRNA expression. Thus, both CWE and CA improved intestinal barrier damage through a decrease in inflammatory cytokines. In addition, CA is a bioactive compound in CWE.

### Protective effect of CWE against DSS-induced colitis in mice

**Fig. 5:**
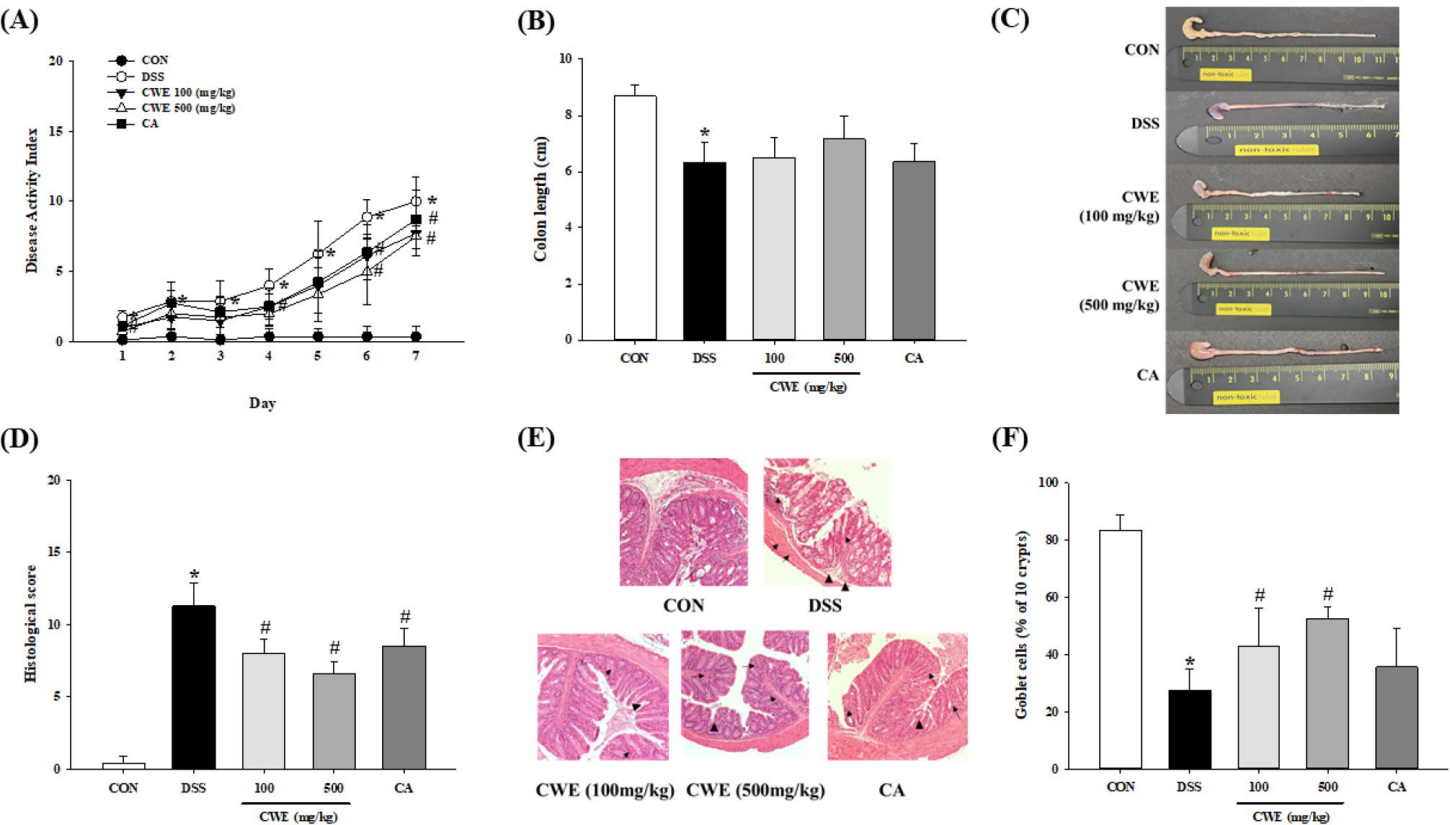
Symptoms in DSS-induced colitis mice improved after CWE treatment. (**A**) Disease activity index, (**B**) colon length, (**C**) representative images of the colon tissue, (**D**) histological scores, (**E**) H&E staining (× 100, demonstrates inflammation in the crypt space (arrow) and regeneration in the crypt space (arrowhead)), and (**F**) goblet cells (%) are shown. All values are shown as the mean ± S.D. Asterisks above the bars indicate significant differences between the CON and DSS groups (Student’s *t* test; *p* < 0.05). Hash marks above the bars indicate significant differences among the DSS-induced groups (Dunnett’s multiple range test; *p* < 0.05). *DSS* dextran sodium sulfate, *CON* control, *DSS* 5% DSS-induced colitis, *CWE 100* DSS + CWE (100 mg/kg B.W.), *CWE 500* DSS + CWE (500 mg/kg B.W.), *CA* DSS + cinnamic acid

The mechanism of DSS-induced colitis has not been clarified. However, a reduction in the mucosa, the death of mucosal cells and the induction of the intestinal immune inflammatory response due to T-cell activity have been investigated in DSS models (Araki et al., 2006; Johansson et al., 2010). To imitate the symptoms of human IBD, such as body weight loss, stool softening, blood in the stool, and rectal bleeding, mice were provided water containing 5% DSS ad libitum, and the survival rate of the mice was recorded for the last week of the experiment. The administration of CWE to mice with DSS-induced colitis strongly exacerbated colitis symptoms and colonic injury. The DAI started to markedly increase from day 5 to 6, the period in which DSS treatment was initiated. Both 100 and 500 mg/kg B.W. of CWE and CA group significantly changed the DAI on day 6–7 compared to that in the DSS group (*p* < 0.01, Fig. 5A). Additionally, the length of the intestine was measured because this length provides insight into inflammatory status. The length of the mouse colons in the control group was 8.7 ± 0.4 cm, while that in the DSS group was 6.3 ± 0.8 cm, which was significantly shorter than that of the control group (*p* < 0.001, Fig. 5B, C). The high-dose CWE group showed enhancement in colon length compared to that of the DSS group, but there were no significantly different. Examination of colon tissue sections by H&E staining confirmed that DSS triggered gut TJ barrier damage, including effects such as crypt loss in the mucosa, inflammatory cell infiltration, and mucosal cell proliferation. The histological score was approximately 4.0-fold higher in the DSS group, while treatment with both CWE and CA reduced the histological score (*p* < 0.001, Fig. 5D, E). The number of goblet cells, which are involved in mucous production, was decreased in the DSS-treated group, whereas the groups treated with low-dose and high-dose CWE showed a significant increase (*p* < 0.001, Fig. 5F).

### Inhibitory effect of CWE on proinflammatory cytokine production in the colon

**Fig. 6:**
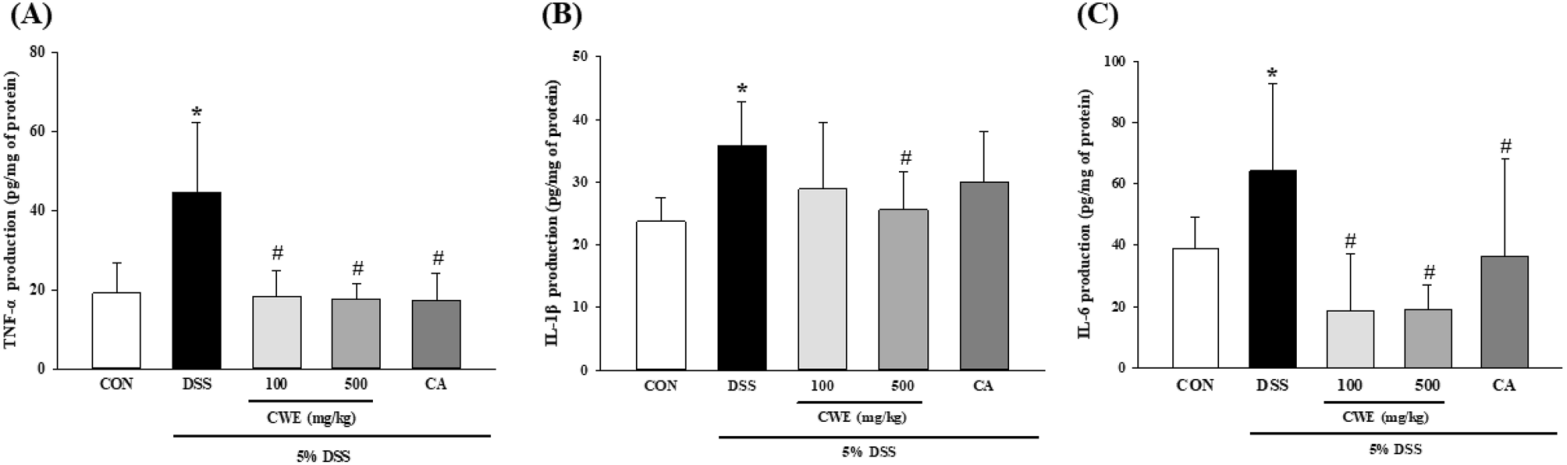
Inhibition of inflammatory cytokine levels in DSS-induced colitis mice after CWE treatment. (**A**) TNF-α, (**B**) IL-1β, and (**C**) IL-6. All values are shown as the mean ± S.D. Asterisks above the bars indicate significant differences between the CON and DSS groups (Student’s *t* test; *p* < 0.05). Hash marks above the bars indicate significant differences among the DSS-induced groups (Dunnett’s multiple range test; *p* < 0.05). *DSS* dextran sodium sulfate, *CON* control, *DSS* 5% DSS-induced colitis, *CWE 100* DSS + CWE (100 mg/kg B.W.), *CWE 500* DSS + CWE (500 mg/kg B.W.), *CA* DSS + cinnamic acid

The concentrations of TNF-α, IL-1β, and IL-6 in the colon from the colitis model mice were quantified. The levels of TNF-α and IL-6 were significantly different between the CON and DSS groups. Treatment with CWE at 100 and 500 mg significantly decreased TNF-α and IL-6 production compared with that in the DSS group. In addition, the CA group showed a significant decrease in TNF-α and IL-6 levels compared to those in the DSS group (*p* < 0.001) (Fig. 6A, C). IL-1β production in the high-dose CWE group was tended to reduce compared to that in the DSS group (Fig. 6B). These results indicated that CWE improved the symptoms of intestinal inflammation and histopathological signs. Based on these results, CWE administration improved barrier damage and inflammation and had a restorative effect.

T lymphocytes and macrophages can secrete cytokines and chemokines due to their activity in the inflammatory response produced by IBD. Immune cell activity causes an imbalance in anti-inflammatory cytokines and pro-inflammatory cytokines by overproducing TNF-α, IL-1β, and IL-6, which are involved in the development of IBD. Elevated IL-1β expression exacerbates IBD, and colitis patients have higher IL-6 levels than healthy people (Kinoshita et al., 1999; Mao et al., 2018). The relationship between TNF-α and IBD was thus explored, as it has been reported that when the concentration of TNF-α increases, chronic colitis is maintained, while when the concentration of TNF-α decreases, the symptoms of chronic colitis are alleviated (Min et al., 2010).

The CWE and CA treatment groups showed significantly enhanced cell permeability, mRNA expression of proinflammatory cytokines, and TJ protein expression. CWE- and CA-treated mice showed improvements in the DAI, histological score, and cytokine concentrations compared with those in the colitis model mice. These results indicated that CA could abrogate damage to the intestinal barrier, suggesting CA is a candidate bioactive component in cinnamon species. CWE contains not only CA but also various phytochemicals, such as cinnamic aldehyde and eugenol. CA is likely only partly responsible for the observed effects of CWE, suggesting additive or synergistic effects with other active ingredients in CWE. Therefore, various bioactive compounds from herbs could have greater beneficial health effects than a single compound (Kim and Kwon, 2011). Thus, CWE contains various phytochemicals and compounds with additive or synergistic effects that should be investigated in further studies.

To the best of our knowledge, this is the first study to investigate how CWE mediates TJ barrier function and exerts anti-inflammatory activity in the gastrointestinal system. Thus, CWE is anticipated to be useful as a beneficial treatment for IBD and other intestinal diseases and to be highly developed as a functional food for improving intestinal health. The limitation of this study is that the precise mechanism of CWE was not evaluated. Further study is needed to confirm the detailed effects of CWE on TJs and its multiple functions, and the levels of various phytochemicals in CWE should be elucidated.

### Supplementary Information

Below is the link to the electronic supplementary material.

Supplementary file1 (DOCX 23 kb)
