# Supplementary material for: Cinnamon (Cinnamomum cassia) hot water extract improves inflammation and tight junctions in the intestine in vitro and in vivo
Source: Food Sci Biotechnol. 2023 Mar 27;32(13):1925–33. doi: 10.1007/s10068-023-01292-3 (PMC10541376; doi:10.1007/s10068-023-01292-3)
Supplement: Supplementary file 1 — Supplementary file1 (DOCX 23 kb) [file 10068_2023_1292_MOESM1_ESM.docx]

**Table S1. HPLC conditions for the determination of cinnamic acid in CWE**

| **Parameters** | **Operation conditions** |
| --- | --- |
| Instrument | Shiseido SI-2 series HPLC system |
| Column | Shiseido Capcellpak C18 UG120 (5㎛, 4.6㎜ x 250㎜) |
| Oven temperature | 40℃ |
| Detector (DAD) | 280 nm |
| Flow rate | 1000 μl/min |
| Injection volume | 10 μl |
| Retention time | 14 min |

**Table S2. Primers used for qRT-PCR analysis in Caco-2 cell**

| **Gene name** | **Forward (5’-3’)** | **Reverse (5’-3’)** |
| --- | --- | --- |
| TNF-α | agcccatgttgtagcaaacc | tctcagctccacgccatt |
| IL-6 | gatgagtacaaaagtcctgatcca | tctgcagccactggttctgt |
| IL-1β | agctacgaatctccgaccac | cgttatcccatgtcgaagaa |
| COX-2 | cttcacgcatcagtttttcaag | tcaccgtaaatatgatttaagtccac |
| iNOS | gaccagtacgtttggcaatg | tttcagcatgaagagcgattt |
| Zonula occludens-1 | aaatttaactaatgtcagactggagga | Tcagcttgtggtgagtaagagg |
| Occludin | gggaacattaaggggattaacct | atgatgcagtttaaattggtattgtt |
| Claudin-1 | caccgtctgtgtttgagca | caaaccaccgcttacagatg |
| Claudin-3 | aacctgcatggactgtgaaa | ggtcaagtattggcggtcac |
| Claudin-4 | aacctgtccccgagagaga | gcaagtgtgagcagaccagt |

**Table S3. Criteria for disease activity index**

| **Score** | **Weight loss (%)** | **Stool consistency** | **Fecal bleeding** |
| --- | --- | --- | --- |
| 0 | <0 | Normal | Normal |
| 1 | 1-5 | - | - |
| 2 | 6-10 | Soft | Slightly bloody |
| 3 | 11-15 | Loose | Bloody |
| 4 | >15 | Diarrhea | Severely bloody |

**Table S4. Criteria for histological score**

| **Feature graded** | **Grade** | **Description** |
| --- | --- | --- |
| Inflammation | 0 | None |
|  | 1 | Slight |
|  | 2 | Moderate |
|  | 3 | Severe |
| Extent | 0 | None |
|  | 1 | Mucosa |
|  | 2 | Mucosa and submucosa |
|  | 3 | Transmural |
| Regeneration | 4 | No tissue repair |
|  | 3 | Surface epithelium not intact |
|  | 2 | Regeneration with crypt depletion |
|  | 1 | Almost complete regeneration |
|  | 0 | Complete regeneration or normal tissue |
| Crypt damage | 0 | None |
|  | 1 | Basal 1/3 damaged |
|  | 2 | Basal 2/3 damaged |
|  | 3 | Only surface epithelium lost |
|  | 4 | Entire crypt and epithelium lost |
| Percent involvement | 1 | 1-25% |
|  | 2 | 26-50% |
|  | 3 | 51-75% |
|  | 4 | 76-100% |
